# Relationship between sleep habits and academic performance in university Nursing students

**DOI:** 10.1186/s12912-021-00635-x

**Published:** 2021-06-17

**Authors:** Juana Inés Gallego-Gómez, María Teresa Rodríguez González-Moro, José Miguel Rodríguez González-Moro, Tomás Vera-Catalán, Serafín Balanza, Agustín Javier Simonelli-Muñoz, José Miguel Rivera-Caravaca

**Affiliations:** 1 Faculty of Health Sciences, Catholic University of Murcia, 30107 Murcia, Spain; 2 Department of Pneumology, Alcalá de Henares, Hospital Universitario Príncipe de Asturias, 28805 Madrid, Spain; 3 Department of Nursing, Physiotherapy and Medicine, Faculty of Health Sciences,, University of Almería, Ctra. Sacramento, s/n 04120 La Cañada de San Urbano, 04007 Almería, Spain; 4 Department of Cardiology, Hospital Clínico Universitario Virgen de la Arrixaca, Universidad de Murcia, Instituto Murciano de Investigación Biosanitaria (IMIB-Arrixaca), CIBERCV, 30120 Murcia, Spain

**Keywords:** Sleep habits, Circadian rhythm, Sleep pattern, Academic performance, Nursing students

## Abstract

**Background:**

Sleep disorders are composed of a group of diseases of increasing prevalence and with social-health implications to be considered a public health problem. Sleep habits and specific sleep behaviors have an influence on the academic success of students. However, the characteristics of sleep and sleep habits of university students as predictors of poor academic performance have been scarcely analyzed. In the present study, we aimed to investigate sleep habits and their influence on academic performance in a cohort of Nursing Degree students.

**Methods:**

This was a cross-sectional and observational study. An anonymous and self-administered questionnaire was used, including different scales such as the ‘Morningness and Eveningness scale’, an author-generated sleep habit questionnaire, and certain variables aimed at studying the socio-familial and academic aspects of the Nursing students. The association of sleep habits and other variables with poor academic performance was investigated by logistic regression. The internal consistency and homogeneity of the ‘sleep habits questionnaire’ was assessed with the Cronbach’s alpha test.

**Results:**

Overall, 401 students (mean age of 22.1 ± 4.9 years, 74.8 % females) from the Nursing Degree were included. The homogeneity of the ‘sleep habits questionnaire’ was appropriate (Cronbach’s alpha = 0.710). Nursing students were characterized by an evening chronotype (20.2 %) and a short sleep pattern. 30.4 % of the Nursing students had bad sleep habits. Regarding the academic performance, 47.9 % of the students showed a poor one. On multivariate logistic regression analysis, a short sleep pattern (adjusted OR = 1.53, 95 % CI 1.01–2.34), bad sleep habits (aOR = 1.76, 95 % CI 1.11–2.79), and age < 25 years (aOR = 2.27, 95 % CI 1.30–3.98) were independently associated with a higher probability of poor academic performance.

**Conclusions:**

Almost 1/3 of the Nursing students were identified as having bad sleep habits, and these students were characterized by an evening chronotype and a short sleep pattern. A short sleep pattern, bad sleep habits, and age < 25 years, were independently associated with a higher risk of poor academic performance. This requires multifactorial approaches and the involvement of all the associated actors: teachers, academic institutions, health institutions, and the people in charge in university residences, among others.

**Supplementary Information:**

The online version contains supplementary material available at 10.1186/s12912-021-00635-x.

## Introduction

Sleep is a complex phenomenon resulting from the interaction between the neuroendocrine system, biological clock and biochemical processes, with environmental, social and cultural aspects that are very relevant in the life stages of adolescence and youth [1]. Indeed, the chronic lack of sleep is a recent worry among adolescents and young university students and it is associated with worse health and clinical outcomes [2, 3].

Among biological factors determining sleep, there are “chronotypes” and sleep patterns. The first term refers to the personal preferences of scheduling the sleep-wake cycle, emphasizing three basic chronotypes: morning (early-risers), and evening (night-owls) and those who are intermediate, defined as those who do not have clear preferences towards any of the extreme schedules for the fulfilling of their activities [4]. The sleep pattern refers to the personal schedule of bedtime and wake-up time. In this sense, a circadian rhythm is a natural, internal process, driven by a circadian clock that repeats roughly every 24 h and regulates the sleep-wake cycle [5].

On the other hand, the sleep habits are in the intersection between biological and cultural values. Endogenous, exogenous or environmental factors are included here, as well as those activities that are developed by the population to induce or maintain sleep, with its study and care becoming a challenge for Nursing [6]. Currently, spontaneous abusive behaviors regarding sleep habits are becoming frequent, leading to a state of chronic sleep deprivation, which translates to fatigue and somnolence during the day [7]. Hence, there is a high prevalence of sleep disorders in university students, especially those that affect the wake-sleep rhythm [2]. For this reason,the interest in establishing relationships between sleep and cognitive processes such as memory, learning ability and motivation, has gained attention during the last years. However, studies that relate sleep with academic problems are scarce, despite previous authors have shown that the reduction of sleep time in teenagers and university students was associated with poor academic performance, accidents and obesity [8, 9]. Since good-quality sleep does not only imply sleeping well at night but also an adequate level of attention during the day for performing different tasks, appropriate sleep has an influence in efficient learning processes in university students [10–12].

Although some scientific evidence has shown a relationship between sleep and low academic performance [13, 14], so far, there are no questionnaires to specifically evaluate sleep habits in Nursing students. Considering that this population has special characteristics, they are mostly young, combine hospital training at the same time they attend classes at the university, they present lifestyles that can negatively influence the academic performance. To study the sleep habits using a specific tool, in addition to analyze the sleep pattern and chronotype, could help to identify students with inappropriate sleep habits for developing interventions to modify these habits. This might have a positive impact on their academic performance and avoid potentially serious negative consequences for their physical and mental health. In the present research, we aimed (a) to design a ‘sleep habits questionnaire’, (b) to analyze the sleep habits, sleep pattern and chronotype, and (c) to investigate sleep habits and their influence on academic performance, in a cohort of Nursing Degree students.

## Methods

### Design and study population

This was an observational, prospective and cross-sectional study involving Nursing students, all of them distributed among the 4 years of the Nursing Degree. There were no inclusion criteria, i.e. all Nursing students were suitable for the study, unless those who did not attend class on the day of data collection, or those who did not wish to participate (from 420 students, 19 refused to participate in the study). The study was fully carried out during the first semester of the 2019–2020 academic year.

### Study Variables

#### Circadian rhythm: the reduced “Horne & Östberg Morningness-Eveningness Questionnaire”

Preferences of schedule for the sleep-wake cycle and its influence on academic performance were assessed using the reduced version of the Horne & Östberg Morningness-Eveningness Questionnaire (rMEQ) proposed by Adan & Almirall [15], translated to Spanish, that is composed of 5 items. The score determines the following five types of schedule: clearly morning type (22–25 points), moderately morning type (18–21 points), no preference (12–17 points), moderately evening type (8–11 points), and clearly evening type (4–7 points). The internal consistency of the circadian rhythm scale assessed using the rMEQ by Adan & Almirall is good, as the scores from all the items are correlated among themselves [15, 16].

#### Sleep habits questionnaire

For the initial design of the sleep habits questionnaire, a panel of 10 voluntary experts was included. This panel was composed of 5 registered nurses and 5 physicians, with a minimum of 5 years of experience in sleep. All of them were interviewed and informed individually about the study. Items composing of the questionnaire were obtained according to the scientific literature and the main factors influencing sleep habits as the discretion of the expert panel [14, 17, 18]. Eleven questions were finally included in a self-reported questionnaire, each ranging from 1 to 4 (never (1), sometimes (2), usually (3), always (4)) (Supplementary file). Sleep habits, including sleep routines, study schedule preference, and napping were also evaluated. The overall score of the questionnaire ranges from 11 to 44 points, with the highest scores indicating the worst sleep habits. As there is no specific cut-off point for this questionnaire, students over the fourth quartile (4Q, i.e. ≥25 points) were categorized as having inappropriate habits. Therefore, these Nursing students were included in the “bad sleeping habits” group.

#### Academic performance

The academic performance was measured by the ratio “failed exams/performed exams” and checked in the student’s academic records. A good academic performance was considered if the final grade of every exam completed during the Nursing Degree was ≥ 5 (in a 0–10 range, where an exam is considered passed if the score is ≥ 5).

### Other variables

Other variables such as gender, age and hours of sleep (sleep pattern), were analyzed. To describe the sleep pattern of the Nursing students, we used the classification described by Miró et al. (2002) [19]. This classification was composed of three categories as a function of the hours slept, so that we found subjects that had a short sleep pattern (< 6 h per day), subjects with a long sleep pattern (≥ 9 h per day), and subjects with an intermediate sleep pattern (6–9 h per day).

#### Ethical considerations

The study protocol was approved by an accredited Ethics Committee (Reference: CE-6191) and was performed in accordance with the ethical standards laid down in the 1964 Declaration of Helsinki. All students were informed and gave consent to participation in the study. The anonymity and confidentiality were guaranteed.

### Statistical analysis

The sample size was calculated by a non-probabilistic sampling technique using Ene 2.0 (GlaxoSmithKline) with a precision ± 5 % and α error = 0.05. This calculation was based on the estimation that the prevalence of bad sleep habits in Nursing students of our university was 30.4 %, which resulted in a minimum sample of 229 subjects.

Categorical variables were expressed as frequencies and percentages. Continuous variables were presented as mean ± standard deviation (SD) or median and interquartile range (IQR), as appropriate.

The Pearson Chi-squared test was used to compare proportions whereas comparison of continuous variables was performed using the Student t test. Correlations between different scales were performed using the Pearson’s correlation test.

In order to investigate if sleep habits and other variables were independently associated with poor academic performance, a logistic regression model (with odds ratios [OR] and two-sided 95 % confidence intervals [CI]) was performed. To measure the internal consistency and homogeneity of the sleep habits questionnaire, the Cronbach’s alpha test was performed.

A *p*-value < 0.05 was accepted as statistically significant. Statistical analyses were performed using SPSS v. 21.0 (SPSS, Inc., Chicago, IL, USA).

## Results

We included 401 Nursing students (100 students from 1st year, 105 from 2nd year, 101 from 3rd year, and 95 from 4th year) in the study. The students were characterized for being predominantly females (300, 74.8 %), with a mean age of 22.1 ± 4.9 years, and the majority of them (88.5 %) were singles.

Sleep habits of the Nursing students were examined using our previously designed (as described in the Methods section) self-reported ‘sleep habits questionnaire’. The homogeneity of the questionnaire was appropriate, with a Cronbach’s alpha value of 0.710. The mean score in the questionnaire was 22.3 ± 3.9, and 30.4 % of the Nursing students had bad sleep habits (i.e. score > 4Q), which were characterized by a clear preference of studying at night, easily lose a night of sleep for work-related or academic tasks that imply staying up late, and showing difficulties in maintaining sleep routines.

Table 1 shows the summarized results for each question of the sleep habits questionnaire.

**Table 1:** Results for each of the variables in the sleep habits questionnaire

***1. Is it difficult for you to maintain a regular schedule for waking up and going to sleep?***
When the students were asked if they usually maintained a regular schedule for waking up and going to sleep, 75.1 % had difficulties in establishing a regular schedule.
***2. Do you change your sleep routines on the weekends and vacation?***
Most (90 %) of the subjects admitted changing their sleep routines during the weekends.
***3. Is it usual for you to take short naps at any point in the day?***
31.2 % (125 subjects) confirmed that they did not take naps, whereas the majority of the students doing so (68.8 %).
***4. Do you easily lose a night of sleep?***
Almost half (47.9 %) of the Nursing students expressed that they lost a night of sleep during the last month.
***5. Do you prefer to study for an exam at night?***
34 % preferred to study at night.
***6. In general, do your work and/or academic activities mean that you have to bed late to achieve your objectives?***
72.5 % of the Nursing students recognized that their work and/or academic activities require going to sleep late.
***7. Do you prefer to sleep late to obtain better study results?***
47.6 % of the students manifested that they preferred going to sleep late to obtain better results from studying. This percentage increased when asked if they believed that going to sleep late improved their academic results.
***8. Do you work, read the newspaper or academic documents just before going to sleep?***
56.4 % worked or read academic documents right before going to bed.
***9. Do you usually listen to music before going to bed?***
47.4 % usually listened to music before going to bed.
***10. Do you usually watch television before going to bed?***
88.8 % often watched television before going to sleep.
***11. Do you go out at night if you have to wake up early the next day to go to class or work?***
50.4 % of the students recognized not having done this on any night during the past month.

The Nursing students in our sample were characterized by an evening chronotype (20.2 %, 81) and a short sleep pattern (i.e. <6 h of sleep daily), with 51.1 % (205) of the students sleeping less than 6 h/day, 42.1 % (169) sleeping 6–9 h/day, and 6.7 % (27) sleeping more than 9 h/day. The mean duration of sleep found in the Nursing students was 6.52 ± 1.4 h.

Of note, most of the Nursing students that had an evening chronotype were < 25 years old (22.2 %, *p* = 0.011). In addition, age showed a positive association with circadian rhythm and as age increased, the students tended to have a predominantly morning chronotype (*R* = 0.223, *p* < 0.001). Nursing students < 25 years of age had also worse sleep habits according to the sleep habits questionnaire than those ≥ 25 years (22.61 ± 3.79 vs. 21.19 ± 4.37, *p* = 0.005). A negative correlation was found between the overall sleep habits questionnaire score and age as a continuous variable (*R* = -0.105, *p* = 0.03).

In addition, 29.5 % of patients that had bad sleep habits (*p* = 0.001), and 23.9 % that had poor academic performance (*p* = 0.020), had also an evening chronotype (Table 2). A significant negative correlation was found between the sleep pattern and sleep habits (*R* = -0.293, *p* < 0.001), and between circadian rhythm and sleep habits, hence Nursing students with good sleep habits have predominantly a morning circadian rhythm (*R* = -0.201, *p* < 0.001).

**Table 2:** Frequency of morningness-eveningness as a function of gender, age, sleep habits and academic achievement

	N (%)			*p*-value
	**Evening** **(*N* = 81)**	**No preference** **(*N* = 291)**	**Morning** **(*N* = 29)**	
**Gender** Male Female	16 (15.8) 65 (21.7)	80 (79.2) 211 (70.3)	5 (5) 24 (8)	0.216
**Age (years)** ≥ 25 < 25	8 (11.1) 73 (22.2)	54 (75) 237 (72.0)	10 (13.9) 19 (5.8)	**0.011**
**Sleep pattern** Intermediate/Long Short	29 (14.8) 52 (25.4)	154 (78.6) 137 (66.8)	13 (6.6) 16 (7.8)	**0.022**
**Sleep habits** Good habits Bad habits	45 (16.1) 36 (29.5)	208 (74.6) 83 (68)	26 (9.3) 3 (2.5)	**0.001**
**Academic performance** Good Poor	35 (16.8) 46 (23.9)	153 (73.2) 138 (71.9)	21 (10) 8 (4.2)	**0.020**

Frequencies calculated starting with sample N = 401

Regarding the academic performance, 93 % (373) of the Nursing students attended all the exams planned, and 47.9 % (192) of the students showed poor academic performance. When we investigated specifically if the sleep habits, as assessed by the ‘sleep habits questionnaire’, influenced the academic performance, we found that 32 % (140) of the Nursing students that had bad sleep habits obtained poor academic results (*p* < 0.001). Those that had the worst academic results were the ones that did not have a regular hour for waking up and going to sleep (2.66 ± 1.03, *p* = 0.031), presented difficulties to maintain the sleep during the night (1.73 ± 0.77, *p* = 0.003), and preferred to study for an exam at night (1.33 ± 0.48, *p* = 0.030), as well as going to bed late to obtain better results (1.46 ± 0.51, *p* = 0.041). Also, those students with poorer academic results where those listening to music before going to bed (1.84 ± 1.10, *p* = 0.007), and going out at night even if they had to get-up early the next day (1.58 ± 0.72, *p* = 0.012). Overall, those Nursing students whose work or academic activities entailed going to bed late to attain their objectives, had the lowest academic performance (2.25 ± 1.01, *p* = 0.001). Lastly, we can confirm that the Nursing students that had better academic performance were the ones who had the best sleep habits. Indeed, the overall ‘sleep habits questionnaire’ score was significantly lower compared to those Nursing students who had poor academic performance (21.91 ± 3.90 vs. 24.18 ± 3.55, *p* < 0.001) (Table 3).

**Table 3:** Association between the scores of each question of the ‘sleep habits questionnaire’ and academic performance

VARIABLE	Academic performance		t	*p*-value
	**Good performance** **Mean (SD)**	**Poor performance** **Mean (SD)**		
ITEM 1. Is it difficult for you to maintain a regular schedule for waking up and going to sleep?	2.38 ± 1.07	2.66 ± 1.03	**-2.14**	**0.031**
ITEM 2. Do you change your sleep routines on the weekends and vacation?	1.90 ± 0.29	1.93 ± 0.26	-0.59	0.554
ITEM 3. Is it usual for you to take short naps at any point in the day?	2.14 ± 0.97	2.26 ± 0.99	-0.97	0.328
ITEM 4. Do you easily lose a night of sleep?	1.73 ± 0.77	2.01 ± 0.80	**-2.95**	**0.003**
ITEM 5. Do you prefer to study for an exam at night?	1.33 ± 0.48	1.46 ± 0.50	**-2.12**	**0.030**
ITEM 6. In general, do your work and/or academic activities mean that you have to bed late to achieve your objectives?	2.25 ± 1.01	2.69 ± 1.02	**-3.48**	**0.001**
ITEM 7. Do you prefer to sleep late to obtain better study results?	1.46 ± 0.51	1.59 ± 0.49	**-1.99**	**0.041**
ITEM 8. Do you work, read the newspaper or academic documents just before going to sleep?	2.06 ± 1.13	2.28 ± 1.13	-1.49	0.136
ITEM 9. Do you usually listen to music before going to bed?	1.84 ± 1.10	2.21 ± 1.12	**-2.69**	**0.007**
ITEM 10. Do you usually watch television before going to bed?	3.24 ± 1.04	3.28 ± 1.06	-0.29	0.774
ITEM 11. Do you go out at night if you have to wake up early the next day to go to class or work?	1.58 ± 0.72	1.81 ± 0.79	**-2.47**	**0.012**
Overall ‘sleep habits questionnaire’ score	21.91 ± 3.90	24.18 ± 3.55	**-4.73**	**< 0.001**

*SD* Standard deviation, *t* Student’s t-test statistic

Finally, the profile of Nursing students with more failed courses was characterized by an evening circadian rhythm (*R* = -0.134, *p* = 0.007), bad sleep habits (*R* = 0.216, *p* < 0.001), and less hours of sleep daily (*R* = -0.211, *p* < 0.001).

To confirm these observations, a multivariate logistic regression analysis was performed. Therefore, a short sleep pattern (adjusted OR = 1.53, 95 % CI 1.01–2.34), bad sleep habits (adjusted OR = 1.76, 95 % CI 1.11–2.79), and age < 25 years (adjusted OR = 2.27, 95 % CI 1.30–3.98) were independently associated with a higher probability of poor academic performance (Table 4).

**Table 4:** Univariate and multivariate analysis of factors associated with the risk of poor academic performance

	Univariate analysis			Multivariate analysis		
	OR	95 % CI	*p*-value	OR	95 % CI	*p*-value
**Sleep pattern**						
Intermediate/Long	Ref.			Ref.		
Short	**1.75**	**1.18–2.59**	**0.006**	**1.53**	**1.01–2.34**	**0.047**
**Circadian rhythm**						
Morning/Intermediate	Ref.			Ref.		
Evening	**0.60**	**0.40–0.89**	**0.011**	1.27	0.76–2.13	0.360
**Sleep habits**						
Good habits	Ref.			Ref.		
Bad habits	**2.10**	**1.36–3.25**	**0.001**	**1.76**	**1.11–2.79**	**0.016**
**Gender**						
Female	Ref.			Ref.		
Male	0.74	0.47–1.17	0.20	1.39	0.87–2.21	0.171
**Age (years)**						
≥ 25	Ref.			Ref.		
< 25	**2.43**	**1.41–4.20**	**0.001**	**2.27**	**1.30–3.98**	**0.004**

*OR* odds Ratio; *CI* confidence interval; *Ref*: reference

## Discussion

Sleep is an excellent indicator of the health status and an element that favors good quality of life [20], but entering university is a change that highly impacts the student in every dimension, including sleep habits [21, 22]. A potential barrier for maximizing performance during the university stage is the irregular sleep schedule, which lead to sleep deficit and high prevalence of somnolence during the day [23]. A review by Shochat et al. (2014) [24] examined the consequences of lack of sleep among Nursing students, and confirmed the relationship between sleep disorders and changes in sleep patterns with a reduced academic performance. Other studies have established that sleep has an integral role in learning and memory consolidation [25, 26]. Therefore, despite some scientific evidence has shown a relationship between sleep and low academic performance [13, 14], the originality of our study was to examine the influence that sleep characteristics exert (chronotypes and sleep patterns), as well as sleep habits of the university population on academic performance.

Overall, the academic performance of our Nursing students was suboptimal. When analyzing how sleep pattern, sleep habits, and circadian rhythms influenced this academic performance, we observed that all of them may be determine factors for learning, as other studies have done [27].

Concerning the sleep pattern, it should be noted that most of the students enrolled in the Nursing Degree slept less than 6 h per day. Of note, our results seem to establish a relationship between the hours slept and the academic performance during the first semester, as gathered from the academic records. This finding is in accordance to observations by other authors in university students from Medicine [9], Pharmacy [2] or Nursing [28], which also showed evidence between the hours slept and the academic achievement. In a previous study, we already observed that university students from the Faculty of Nursing attributed the hours slept with academic performance [29]. Indeed, it should be highlighted that chronic lack of sleep is not only associated with alterations of attention and academic performance, but also to a series of adverse consequences for health such as risky behaviors, depression, anxiety, alterations in social relations, and obesity, among others [30].

In addition, our study has evidenced how the sleep habits directly influenced the academic performance of these Nursing students, and approximately 1/3 of the students with bad sleep habits obtained poor academic results. Certainly, the sleep pattern and inadequate sleep habits could be related. Good sleep hygiene includes aspects such as a regular sleep-wake schedule, adequate environment, avoiding stimulating activities before going to bed, and limiting the use of technology in bed or immediately before going to bed. In the present study, 30.4 % of the students had bad sleep habits, characterized by having a clear preference for studying at night, often losing a night of sleep for work or academic activities that imply go to bed late, and show difficulties in maintaining sleep routines. An important proportion of our Nursing degree students declared that they watched television, listened to music, worked or read academic documents during the last hour before going to bed. In this sense, LeBourgeois et al. (2017) [31] have described the university population as great consumers of technology, and have associated the frequent use of technology before going to bed with problems to sleep and daytime somnolence.

Finally, age was another factor that should be considered in the analysis of sleep habits. According to our results, the Nursing students that were < 25 years of age had the worst sleep habits and used to have more difficulties in maintaining sleep routines, modifying them on the weekends and holidays, preferring to stay up late to obtain better study results, and going out at night without considering that they had to get up early. As other studies [21], we observed that social activities were a priority in the life of the university adolescents and the substituting of hours of sleep for enjoying and sharing activities with friends and classmates did not constitute a problem for them. These behaviors were added to the physiological delay of the start of sleep that is typical in this stage of life and might unleash deprivation or a chronic deficit of sleep, maintained throughout the entire week. The students then tried to compensate for this lack of sleep by increasing their hours of sleep during the weekend. We agree with previous studies that this circumstance, far from minimizing or compensating the effects of sleep deprivation, aggravates them, worsening the pattern and the quality of sleep of the students [22].

Further, we found an association between age and circadian type. We observed that most of the university students with evening chronotypes were aged < 25, had bad sleep habits, and a poor academic performance. Physiologically, adolescents and adults tend to have delayed circadian preferences and are “lovers of the night” [23]. In our study, 20.2 % of students had an evening chronotype, which is lower than that reported in other studies, where 59 % of the students between 18 and 29 years of age described themselves as night owls [32]. Our results also showed a clear normalization of the evening behaviors of the students. These data are in agreement with other authors who highlighted the influence exerted by the aforementioned normalization of evening habits among the youth on the quality of sleep, leading to a medium to long-term sleep deficit [20]. As Crowley et al. (2018) [33], we think that evening behavior leads to asynchrony between the biological rhythm and the social life of the student, having negative consequences on the academic performance. However, how this really affects academic results requires extending researches, since the circadian rhythm was not significantly associated with academic performance.

The results of this study evidence the need to seriously take into consideration the sleep deficits that are associated with inadequate sleep habits, with the aim of developing preventative and educational initiatives to improve the sleep habits of the university population. The challenge ahead starts with the social awareness of the importance of having good-quality sleep since many times, adequate knowledge about sleep does not translate into a change of sleep habits [23].

### Limitations

Some limitations should be noted. Due to the cross-sectional design of the study, we could not establish an exact causal relationship between sleep pattern and academic performance. In addition, it should be note that the ‘sleep habits questionnaire’ is a subjective questionnaire, and therefore the result could be biased if the student did not answer honestly. Another limitation is the difficulty in conceptualizing academic performance, due to its complex and multi-causal character, where many factors intervene. The factors include attitudes, habits, the character of the staff, methodologies, family environment, organization of the educational system, socio-economic condition, as well as other social, economic, and psychological aspects [34]. Finally, the study was conducted only in Nursing students, so our results must be prospectively validated in University students from a larger variety of academic sectors. Similarly, this study was conducted in a single University, so more studies involving other Universities are also necessary. Despite these circumstances, we believe that our hypothesis that the duration of sleep could lead to better academic performance is based on current scientific data.

## Conclusions

Using the 11-item ‘sleep habits questionnaire’, 30.4 % of the Nursing students were identified as having bad sleep habits. In addition, Nursing students included in this research were characterized by an evening chronotype and a short sleep pattern. Regarding academic performance, half of the Nursing students showed a poor one. A short sleep pattern, bad sleep habits, and younger age, were independently associated with a higher risk of poor academic performance. This requires multifactorial approaches and the involvement of all the associated actors: teachers, academic institutions, health institutions, and the people in charge in university residences, among others.

## Supplementary Information

**Additional file 1:**

## Data Availability

The datasets used and/or analysed during the current study are available from the corresponding author on reasonable request.
