# Supplementary material for: Relationship between sleep habits and academic performance in university Nursing students
Source: BMC Nurs. 2021 Jun 17;20:100. doi: 10.1186/s12912-021-00635-x (PMC8212524; doi:10.1186/s12912-021-00635-x)
Supplement: Supplementary file 1 — Additional file 1: [file 12912_2021_635_MOESM1_ESM.docx]

**Relationship between sleep habits and academic performance in university students**

Juana Inés Gallego-Gómez ^1^, María Teresa Rodríguez González-Moro^1^, José Miguel Rodríguez González-Moro^2^, Tomás Vera-Catalán^1^, Serafín Balanza ^1^, Agustín Javier Simonelli-Muñoz ^3*^, and José Miguel Rivera-Caravaca^4^.

^1^Faculty of Health Sciences, Catholic University of Murcia, 30107 Murcia, Spain.

^2^Department of Pneumology, Hospital Universitario Príncipe de Asturias, Alcalá de Henares, 28805 Madrid, Spain.

^3^Department of Nursing, Physiotherapy and Medicine, University of Almería, 04007 Almería, Spain. *Corresponding Author (sma147@ual.es).

^4^Department of Cardiology, Hospital Clínico Universitario Virgen de la Arrixaca, Instituto Murciano de Investigación Biosanitaria (IMIB-Arrixaca), CIBERCV, 30005 Murcia, Spain.

**Version of the Sleep habits questionnaire (English translation).**

|  | Never | Sometimes | Usually | Always |
| --- | --- | --- | --- | --- |
| ITEM 1. Is it difficult for you to maintain a regular schedule for waking up and going to sleep? |  |  |  |  |
| ITEM 2. Do you change your sleep routines on the weekends and vacation? |  |  |  |  |
| ITEM 3. Is it usual for you to take short naps at any point in the day? |  |  |  |  |
| ITEM 4. Do you easily lose a night of sleep? |  |  |  |  |
| ITEM 5. Do you prefer to study for an exam at night? |  |  |  |  |
| ITEM 6. In general, do your work and/or academic activities mean that you have to bed late to achieve your objectives? |  |  |  |  |
| ITEM 7. Do you prefer to sleep late to obtain better study results? |  |  |  |  |
| ITEM 8. Do you work, read the newspaper or academic documents just before going to sleep? |  |  |  |  |
| ITEM 9. Do you usually listen to music before going to bed? |  |  |  |  |
| ITEM 10. Do you usually watch television before going to bed? |  |  |  |  |
| ITEM 11. Do you go out at night if you have to wake up early the next day to go to class or work? |  |  |  |  |
